# Formalizing method choice: a data-driven selection of mechanistic models for predicting human heart drug partitioning

**DOI:** 10.1007/s10928-026-10057-4

**Published:** 2026-09-25

**Authors:** Seweryn Ulaszek, Bartek Lisowski, Monika Jesionek, Barbara Wiśniowska, Sebastian Polak

**Affiliations:** 1 Chair of Pharmaceutical Technology and Biopharmaceutics, Faculty of Pharmacy, Jagiellonian University Medical College, Medyczna 9, Kraków, 30-688 Poland; 2 Doctoral School of Medical and Health Sciences, Jagiellonian University Medical College, Kraków, Poland; 3 Department of Social Pharmacy, Faculty of Pharmacy, Jagiellonian University Medical College, Medyczna 9 Street, Krakow, 30-688 Poland; 4 Certara Predictive Technologies (a Certara Company), Level 2-Acero, 1 Concourse Way, Sheffield, S1 2BJ UK

**Keywords:** Partition coefficient, Heart, Drug distribution, Pharmacokinetics

## Abstract

We developed a workflow based on a human-derived dataset, aiming to predict the human heart-to-plasma partition coefficient, $\:{K}_{p}$. The workflow combines established mechanistic tissue-composition models with an AI/ML-based selector that supports compound-specific selection among mechanistic heart $\:{K}_{p}$ prediction methods. Three published mechanistic approaches were implemented and parameterized in a harmonized manner consistent with the described methodologies to generate baseline $\:{K}_{p}$ predictions. The ML-based selector was trained using physicochemical, ADME, and pharmacokinetic descriptors to recognize compound-specific differences in model performance. This hybrid strategy preserves mechanistic interpretability while using empirical pattern recognition to formalize a priori model choice across compounds. In internal cross-validation, the selector reduced extreme outliers and provided a reproducible, data-driven rule for compound-specific method selection, although it did not materially outperform the strongest standalone mechanistic baseline in numerical error. Although no individual mechanistic method performed consistently across the full chemical space, the Poulin–Theil approach showed the best overall performance on human whole-heart data. The workflow was also applied prospectively to transthyretin stabilizers, for which reliable heart $\:{K}_{p}$ estimates may support further mechanistic modelling of cardiac drug exposure and downstream pharmacological effects. Together, these findings suggest that combining mechanistic calculators with an AI/ML-based selector may provide a practical basis for a priori, compound-specific method selection, reducing reliance on subjective choice of a single mechanistic approach.

**Supplementary Information:**

The online version contains supplementary material available at https://doi.org/10.1007/s10928-026-10057-4.

## Introduction

Heart tissue penetration is an important determinant of cardiac drug exposure and is therefore relevant to physiologically based pharmacokinetic (PBPK) modelling. Within PBPK frameworks, tissue distribution is commonly parameterized using tissue-to-plasma partition coefficients ($\:{K}_{p}$), which quantify the steady-state concentration ratio between tissue and plasma [1]. Over the past two decades, multiple mechanistic tissue-composition-based approaches have been proposed to estimate $\:{K}_{p}$values from compound properties such as lipophilicity, pKa, and fraction unbound in plasma together with tissue composition parameters, including the methods of Poulin–Theil, Rodgers–Rowland, and Schmitt [1–7]. In practice, however, predictive performance varies across compounds and tissues, and comparative evaluations indicate that no single published method for estimating $\:{K}_{p}\:$is uniformly optimal when applied broadly [8]. This suggests that $\:{K}_{p}\:$estimation should be considered not only a calculation problem, but also a model-selection problem.

Yun and colleagues proposed a decision-tree–based framework for selecting, for a given compound and tissue, the $\:{K}_{p}$ prediction algorithm most likely to perform best among multiple published methods [9]. Their work demonstrated the feasibility of structured, data-driven algorithm selection for tissue distribution modelling across multiple organs. Building on that concept, the present study focuses on human whole-heart $\:{K}_{p}\:$(denoted throughout this publication as $\:{K}_{p,\mathrm{h}\mathrm{e}\mathrm{a}\mathrm{r}\mathrm{t}}$) through a selector framework trained on human heart distribution data and evaluated across multiple empirical routines for compound-specific method selection [10]. The resulting workflow is intended to support cardiac exposure modelling, including prospective application to transthyretin stabilizers.

**Fig. 1:**
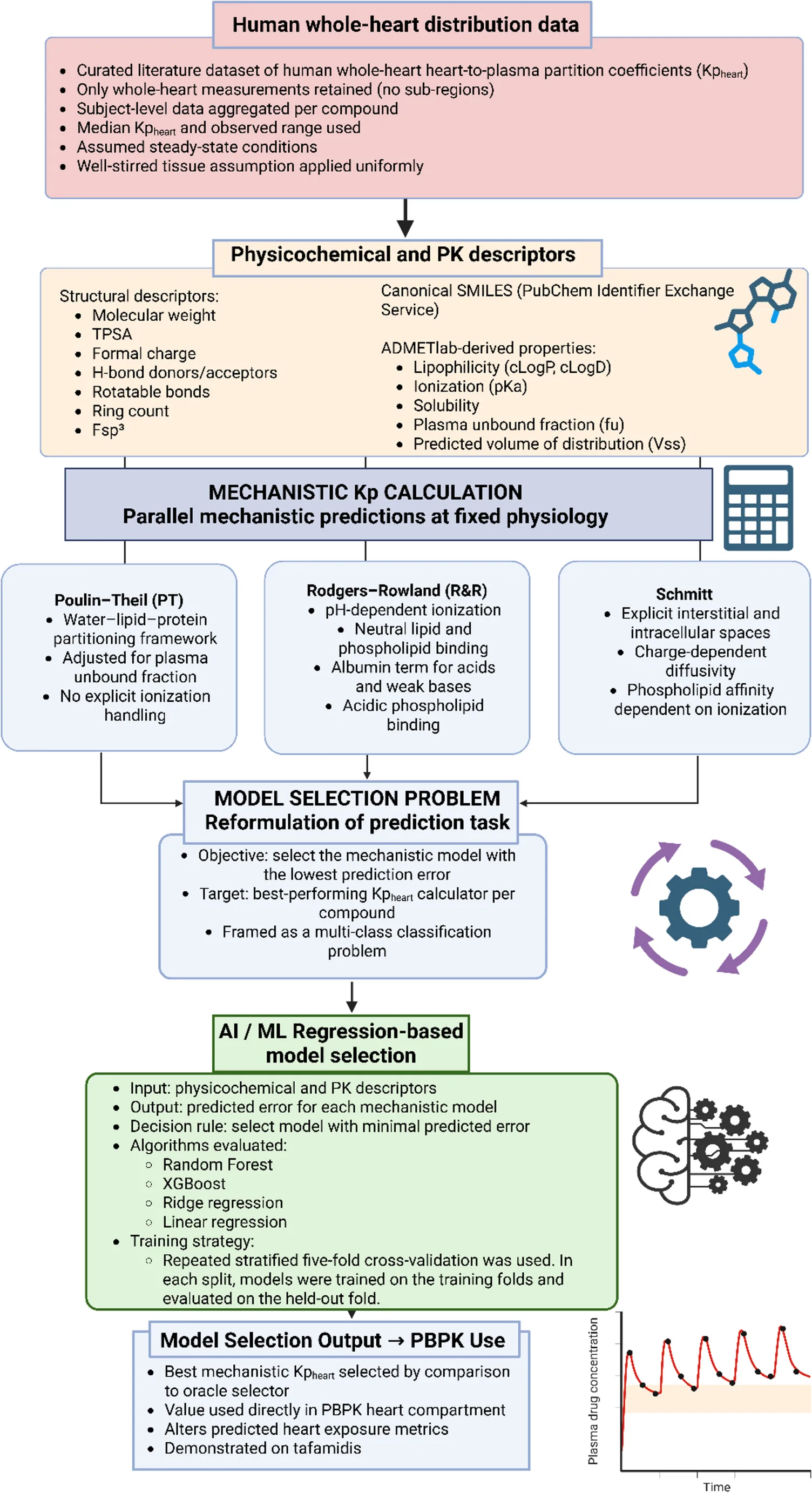
Diagram of the proposed workflow

Accordingly, the aim of the current work was to develop and test a reproducible framework for predicting $\:{K}_{p,\mathrm{h}\mathrm{e}\mathrm{a}\mathrm{r}\mathrm{t}}\:$using only the data on human whole-heart drug concentration (Fig. 1). Three mechanistic models (Poulin–Theil, Rodgers–Rowland, and Schmitt) were implemented using equations and assumptions derived from original publications [1, 3, 4]. No recalibration of these equations was performed. The objective was to assess whether a structured selection layer improves heart-specific predictive performance when evaluated within a human heart dataset.

As a prospective translational application, the workflow was further applied to transthyretin stabilizers. For this drug class, heart $\:{K}_{p}\:$is relevant because it can influence estimated cardiac exposure and can therefore support subsequent mechanistic modeling of drug effects in the heart. Tafamidis was selected as an illustrative example because a previously developed PBPK model was available [11], allowing the predicted $\:{K}_{p,\mathrm{h}\mathrm{e}\mathrm{a}\mathrm{r}\mathrm{t}}\:$value to be propagated into an explicit heart compartment and used to examine its impact on organ-level exposure predictions.

## Theoretical

The theoretical framework of this study is based on three published tissue-composition models implemented to calculate $\:{K}_{p,\mathrm{h}\mathrm{e}\mathrm{a}\mathrm{r}\mathrm{t}}$: Poulin–Theil (PT), Rodgers–Rowland (R&R), and Schmitt. These models estimate heart-to-plasma partitioning from compound-specific physicochemical properties, plasma binding, ionization state, and physiological tissue-composition parameters. They were selected because they represent established, physiologically interpretable frameworks for tissue partitioning and could be applied systematically across the curated dataset using publicly available or inferable compound-specific inputs. Although all three models predict the same endpoint, $\:{K}_{p,\mathrm{h}\mathrm{e}\mathrm{a}\mathrm{r}\mathrm{t}}$, they differ in how they formalize lipid partitioning, tissue water distribution, plasma and tissue protein binding, ionization-dependent distribution, and phospholipid interactions. Therefore, the equations below are presented to define the mechanistic assumptions underlying each candidate $\:{K}_{p,\mathrm{h}\mathrm{e}\mathrm{a}\mathrm{r}\mathrm{t}}$ calculator before their empirical comparison and AI/ML-based selection. All three models were implemented using equations and assumptions derived from their published approaches, without recalibration of the original mechanistic structure.

### Poulin–Theil (PT)

The heart-to-plasma partition coefficient was calculated using a Berezhkovskiy-corrected partition expression [1, 12, 13]:

1 $$\:\begin{array}{c}{K}_{p,\mathrm{heart}}=\frac{\left({f}_{NL,t}+0.3{f}_{NP,t}\right)P+0.7{f}_{NP,t}+{V}_{W,t}/{f}_{ut}}{\left({f}_{NL,p}+0.3{f}_{NP,p}\right)P+0.7{f}_{NP,p}+{V}_{W,p}/{f}_{up}\:}\end{array}$$

where $\:{f}_{NL,t}$ and $\:{f}_{NP,t}$ are the tissue neutral lipid and neutral phospholipid fractions, $\:{f}_{NL,p}$ and $\:{f}_{NP,p}$ are the corresponding plasma fractions, $\:{V}_{W,t}$ and $\:{V}_{W,p}$ are the tissue and plasma water terms, $\:{f}_{ut}$ is the tissue unbound fraction, $\:{f}_{up}$ is the plasma unbound fraction, and * P* is the octanol to water partition coefficient of the unionized compound. The latter was calculated as:

2 $$\:\begin{array}{c}P={10}^{\mathrm{log}P}\end{array}$$

In the present workflow, the plasma and tissue binding terms were defined as:

3 $$\:\begin{array}{c}{B}_{p}=\frac{1-{f}_{up}}{{f}_{up}}\:\:and\end{array}$$

4 $$\:\begin{array}{c}{B}_{t}=0.5{B}_{p}\end{array}$$

and the tissue unbound fraction was then estimated as:

5 $$\:\begin{array}{c}{f}_{ut}=\frac{1}{1+{B}_{t}}\end{array}$$

### Rodgers–Rowland (R&R)

For acids and weak bases, the unbound tissue-to-plasma water partition coefficient was represented as the sum of intracellular water, extracellular water, lipid, and extracellular protein-binding contributions [3]:

6 $$\:\begin{array}{c}K_{pu}=f_{EW}+\frac XYf_{IW}+\\\frac{Pf_{NL}+\left(0.3P+0.7\right)f_{NP}}Y+K_{aPR}{\left[PR\right]}_T\end{array}$$

and the corresponding tissue-to-plasma partition coefficient was calculated as:

7 $$\:\begin{array}{c}{K}_{p}={K}_{pu}{f}_{up}\end{array}$$

where $\:{f}_{EW}\:$and $\:{f}_{IW\:}$are the extracellular and intracellular water fractions, $\:{f}_{NL}$and $\:{f}_{NP}$are the neutral lipid and neutral phospholipid fractions, $\:{K}_{aPR}\:$is the extracellular protein association term, $\:\left[PR{]}_{T}\right.\:$is the tissue extracellular protein term, and * X*and * Y*are ionization terms.

For acids, the ionization terms were defined as:

8 $$\:\begin{array}{c}X=1+{10}^{\left(p{H}_{t}-p{K}_{a}\right)}\end{array}$$

9 $$\:\begin{array}{c}Y=1+{10}^{\left(p{H}_{p}-p{K}_{a}\right)}\end{array}$$

For weak bases, the ionization terms were defined as:

10 $$\:\begin{array}{c}X=1+{10}^{\left(p{K}_{a}-p{H}_{t}\right)}\end{array}$$

11 $$\:\begin{array}{c}Y=1+{10}^{\left(p{K}_{a}-p{H}_{p}\right)}\end{array}$$

For strong bases, the Rodgers formulation additionally included acidic phospholipid binding [2]:

12 $$\begin{array}{c}K_{pu}=f_{EW}+\frac XYf_{IW}+\\\frac{Pf_{NL}+\left(0.3P+0.7\right)f_{NP}}Y+\frac{K_{aAP}{\left[AP^-\right]}_T\hspace{0.17em}10^{\left(pK_a-pH_t\right)}}Y\end{array}$$

where $\:{K}_{aAP}$ is the acidic phospholipid association term and $\:\left[A{P}^{-}{]}_{T}\right.$ is the tissue acidic phospholipid term. The corresponding tissue-to-plasma partition coefficient was then calculated as:

13 $$\:\begin{array}{c}{K}_{p}={K}_{pu}{f}_{up}\end{array}$$

For strong bases, the ionization terms were defined as:

14 $$\:\begin{array}{c}X=1+{10}^{\left(p{K}_{a}-p{H}_{t}\right)}\end{array}$$

15 $$\:\begin{array}{c}Y=1+{10}^{\left(p{K}_{a}-p{H}_{p}\right)}\end{array}$$

The acidic phospholipid affinity term was estimated from blood-cell partitioning logic according to:

16 $$\:\begin{array}{c}{K}_{pu,BC}=\frac{B:P+H-1}{H{f}_{up}}\end{array}$$

where $\:B:P\:$is the blood-to-plasma ratio, $\:H\:$is the hematocrit, and $\:{K}_{pu,BC}\:\:$is the unbound blood-cell-to-plasma partition coefficient. In the present workflow, compounds with $\:p{K}_{a}<7\:$were treated as weak bases and compounds with $\:p{K}_{a}\ge\:7\:$as strong bases. This implementation follows the Rodgers framework for moderate-to-strong bases together with its extension to acids and weak bases [2, 3].

###  Schmitt

The Schmitt model was implemented as an interstitial/cellular tissue-composition framework. Tissue-to-plasma partitioning was expressed as [4]:

17 $$\:\begin{array}{c}{K}_{t:p}=\left(\frac{{F}_{int}}{{f}_{u,int}}+\frac{{F}_{cell}}{{f}_{u,cell}}\right){f}_{u,p}\end{array}$$

where $\:{F}_{int}$ and $\:{F}_{cell}$ are the interstitial and cellular volume fractions, and $\:{f}_{u,int}$, $\:{f}_{u,cell}$, and $\:{f}_{u,p}$ are the unbound fractions in interstitial space, cellular space, and plasma, respectively.

The interstitial unbound fraction was derived from plasma binding as:

18 $$\:\begin{array}{c}\frac{1}{{f}_{u,int}}={F}_{W,int}+\frac{{F}_{P,int}}{{F}_{P,pl}}\left(\frac{1}{{f}_{u,p}}-{F}_{W,pl}\right)\end{array}$$

where $\:{F}_{W,int}$ and $\:{F}_{W,pl}$ are the water fractions in interstitium and plasma, and $\:{F}_{P,int}$ and $\:{F}_{P,pl}$ are the corresponding protein fractions.

The cellular unbound fraction was calculated from the summed contributions of water, neutral lipid, neutral phospholipid, acidic phospholipid, and protein:

19 $$\begin{array}{c}\frac1{f_{u,cell}}=F_W+K_{nL}F_{nL}+K_{nPL}\\F_{nPL}+K_{aPL}F_{aPL}+K_PF_P\end{array}$$

where $\:{F}_{W}$, $\:{F}_{nL}$, $\:{F}_{nPL}$, $\:{F}_{aPL}$, and $\:{F}_{P}$ are the respective cellular fractions, and $\:{K}_{nL}$, $\:{K}_{nPL}$, $\:{K}_{aPL}$, and $\:{K}_{P}$ are the corresponding partition coefficients.

When direct neutral phospholipid partitioning data were unavailable, $\:{K}_{nPL}$ was estimated from lipophilicity as:

20 $$\:\begin{array}{c}{K}_{nPL}={10}^{\mathrm{log}P}\end{array}$$

following tissue-composition framework proposed by Schmitt in [4].

## Methods

Human heart physiological and tissue-composition parameters required for implementation of the mechanistic models were taken from the original model publications and incorporated as fixed values within the unified workflow [1–4]. Parameters representing physiological conditions are listed in Online Resource 2, and the overall workflow is shown in Fig. 1.

### Data curation

Training data were drawn from a curated database of publicly available $\:{K}_{p,heart}$ values [14]. Only whole-heart measurements were retained, whereas records referring to specific anatomical heart regions were excluded. Subject-level entries were then aggregated so that each drug contributed a single record defined by the median $\:{K}_{p,\mathrm{h}\mathrm{e}\mathrm{a}\mathrm{r}\mathrm{t}}$ together with its observed range (minimum–maximum). As steady-state conditions were rarely explicitly reported in the original studies, all observations were treated under a steady-state assumption for the purpose of consistent comparative model development. Each compound was assigned an isomeric SMILES string using the PubChem Identifier Exchange Service and subsequently queried in ADMETlab 3.0 [15]. From this source, routinely obtainable parameters relevant to drug distribution were assembled together with the core inputs required by the mechanistic calculators [16, 17]. Accordingly, the compound-level physicochemical, ADME, and pharmacokinetic descriptors used in the workflow were computationally predicted or database-derived descriptors obtained through ADMETlab 3.0, rather than systematically measured experimental input values. These included predicted descriptors describing lipophilicity and solubility (e.g., clogP, clogD at physiological pH, and clogS), ionization (acidic and basic pKa), plasma unbound fraction, and empirical distribution metrics such as predicted steady-state volume of distribution ($\:{V}_{d,ss}$). In addition, size- and polarity-related descriptors associated with tissue partitioning were included, such as molecular weight, topological polar surface area (TPSA), formal charge, numbers of hydrogen-bond acceptors and donors, rotatable bond count, ring count, $\:{F}_{s{p}^{3}}$, and related structural descriptors [18, 19]. Collectively, these features captured compound properties relevant to both the empirical selector and the mechanistic models. Records lacking the information required for mechanistic prediction or selector-model training were excluded from the dataset. The complete list of ADMETlab 3.0 descriptors used for model training, cross-validation dataset construction, and prospective tafamidis $\:{K}_{p,\mathrm{h}\mathrm{e}\mathrm{a}\mathrm{r}\mathrm{t}}$ prediction is provided in the supplementary materials (Online Resource 1). Blood-to-plasma partition ratios for each drug were predicted with built-in Simcyp v25 predictor, using logP, pKa, fu with assumed plasma pH of 7.4 and haematocrit of 45% [20].

### Model selector

For each compound, $\:{K}_{p,\mathrm{h}\mathrm{e}\mathrm{a}\mathrm{r}\mathrm{t}}\:$was calculated using the three mechanistic models described above. These predicted values were then compared with the observed median $\:{K}_{p,\mathrm{h}\mathrm{e}\mathrm{a}\mathrm{r}\mathrm{t}}$.

The model-selection problem was formulated as a regression-based selection task. Instead of directly assigning compounds to mechanistic-method classes, separate regression models were trained to estimate the expected log-scale prediction error associated with each mechanistic approach. For each compound and mechanistic model * m*, the absolute log-error was defined as:

21 $$\:\begin{array}{c}e_m=\left|log\left(Kp,m+\epsilon\:\right)-log\left(Kp,obs+\epsilon\:\right)\right|\end{array}$$

where $\:{K}_{p,m}$ denotes the partition coefficient predicted by model * m*, $\:{K}_{p,\mathrm{o}\mathrm{b}\mathrm{s}}$ denotes the observed median $\:{K}_{p,\mathrm{h}\mathrm{e}\mathrm{a}\mathrm{r}\mathrm{t}}$, and $\:\epsilon\:={10}^{-12}$ is a small constant introduced for numerical stability.

The selector was trained to predict $\:{\widehat{e}}_{PT}$, $\:{\widehat{e}}_{RR}$, and $\:{\widehat{e}}_{Sch}$ from compound descriptors, and the final mechanistic method was chosen as the one associated with the lowest predicted error:

22 $$\:\begin{array}{c}\widehat m=\mathrm{arg}\underset m{\mathrm{min}}\widehat{e_m}\end{array}$$

This formulation preserves interpretability, because the mechanistic equations remain unchanged, while allowing data-driven adjustment for systematic method-specific differences in predictive performance.

### Cross-validation and preprocessing

Repeated stratified 5-fold cross-validation with 10 repetitions was used for internal training and evaluation of the selector. Stratification was based on the oracle class, defined for each compound as the mechanistic method with the lowest observed absolute log-error. Within each split, preprocessing was performed using the training fold only and then transferred unchanged to the corresponding test fold. Numeric predictors were imputed using training-fold medians, whereas categorical predictors were encoded using training-fold factor levels, with unseen levels assigned to an “Unknown” category.

### Selector backends

Four regression backends were evaluated for predicting method-specific log-errors: random forest (RF), extreme gradient boosting (XGBoost), ridge regression (RIDGE), and ordinary linear regression (LM).

### Random forest

Random forest is an ensemble learning method that aggregates predictions from multiple decision trees trained on bootstrap-resampled datasets. At each split, a random subset of predictors is considered, reducing correlation between trees and improving generalization. This approach captures nonlinear relationships and high-order interactions without requiring explicit feature engineering and is generally robust to mixed descriptor scales and heterogeneous predictor sets. In the present study, random forest was implemented in regression mode to predict the expected log-error associated with each mechanistic $\:{K}_{p}$ method [21].

### Extreme gradient boosting

XGBoost is a gradient boosting algorithm that sequentially constructs decision trees to minimize a specified loss function, here the squared error loss. Each new tree is fitted to the residual structure not captured by the preceding ensemble, allowing efficient representation of complex nonlinear patterns. Regularization, shrinkage, and row- and column-subsampling contribute to improved generalization and reduced overfitting risk. In the present workflow, XGBoost was implemented in regression mode to predict method-specific log-errors from the encoded descriptor matrix [22].

### Ridge regression

Ridge regression is a linear model with $\:{L}_{2}$-regularization, obtained by minimizing squared error together with a penalty on coefficient magnitude. This penalization stabilizes estimation in the presence of correlated predictors and limits variance inflation in descriptor-rich settings. In the current study, predictors were standardized before model fitting, and ridge regression served as a regularized linear baseline for method-specific error prediction [23].

### Ordinary linear regression (LM)

Ordinary linear regression was included as a transparent unregularized baseline for method-specific error prediction. The model estimates a linear relationship between the encoded compound descriptors and the observed log-scale prediction error for each mechanistic $\:{K}_{p}$ method by minimizing the residual sum of squares. This use is consistent with previous applications of linear-model frameworks to characterize predictive errors of complex mechanistic models as a function of system descriptors, although in the present workflow the predicted errors were further used to support selection among competing mechanistic $\:{K}_{p}$ methods. In contrast to ridge regression, LM does not apply coefficient shrinkage and is therefore more sensitive to multicollinearity, high-dimensional descriptor sets, and influential observations. However, its inclusion provides a simple and interpretable reference model for assessing whether more flexible machine-learning approaches, such as random forest or XGBoost, provide added value beyond a purely linear descriptor–error relationship. In the present workflow, LM was implemented in regression mode to predict method-specific log-errors from the encoded descriptor matrix [24].

### Evaluation strategy

Selector development and backend comparison were conducted using repeated stratified five-fold cross-validation with 10 repetitions. Stratification was based on the oracle class, defined retrospectively for each compound as the mechanistic method associated with the lowest observed absolute log-error. The oracle was used exclusively as a reference for evaluating selector agreement and was not available prospectively.

In each split, models were trained on four folds and evaluated on the remaining fold. The term “evaluation fold” is used here to distinguish these cross-validation evaluations from an independent final test set, which was not used in the present analysis. Within each split, preprocessing was performed using the training fold only and then transferred unchanged to the corresponding evaluation fold in order to prevent information leakage. Numeric predictors were imputed using training-fold medians, whereas categorical predictors were encoded using factor levels defined in the training fold, with previously unseen levels mapped to an “Unknown” category.

Out-of-fold (OOF) predictions were then pooled across all repeated cross-validation runs. An OOF prediction was defined as a prediction generated for a compound while that compound was held out from model training and included only in the evaluation fold. Thus, pooled OOF performance summarizes held-out predictions across all folds and repetitions and provides an aggregate cross-validation-based estimate of selector performance.

Selector performance was evaluated by comparing the predicted best mechanistic method with the oracle class across all cross-validation evaluation folds. The primary decision metric was balanced accuracy, defined as the mean recall across the three oracle classes: Poulin–Theil, Rodgers–Rowland, and Schmitt. For a given oracle class, recall was calculated as the proportion of compounds assigned to that oracle class for which the selector correctly identified the same mechanistic method. This metric was selected to reduce sensitivity to oracle-class imbalance.

Backend ranking was based primarily on median cross-validation balanced accuracy against the oracle, with median Cohen’s κ and median logMAE used as secondary ranking criteria. After backend selection, the chosen random forest selector was refit on the full labelled dataset and applied to tafamidis and acoramidis as an illustrative prospective use case.

As an illustrative prospective use case, tafamidis $\:{K}_{p,\mathrm{h}\mathrm{e}\mathrm{a}\mathrm{r}\mathrm{t}}$ estimates obtained with Poulin–Theil, Rodgers–Rowland, and Schmitt were separately implemented in a previously developed whole-body tafamidis PBPK model [25]. The simulations were performed under the same dosing scenario, and systemic plasma and heart exposures were summarized over the day-7 dosing interval from 144 to 168 h after treatment initiation. The heart compartment was treated as distributive only, without intrinsic clearance.

Additional selector-performance metrics included overall selection accuracy, Cohen’s κ, confusion matrices, and a binomial test against a random-choice baseline of 1/3. Overall accuracy quantified the proportion of compounds for which the selector chose the same mechanistic model as the oracle [26].

Cohen’s $\:\kappa\:\:$quantified chance-corrected agreement between the selector and the oracle [27].

Confusion matrices were used to summarize class-specific agreement and misclassification structure [28]. The binomial test assessed whether the observed selection accuracy exceeded that expected under random choice among three mechanistic models.

To quantify numerical agreement between selected and observed $\:{K}_{p,\mathrm{h}\mathrm{e}\mathrm{a}\mathrm{r}\mathrm{t}}\:$values, regression-based performance measures were also calculated, including log-scale mean absolute error (logMAE), mean absolute error (MAE), root mean squared error (RMSE), and geometric mean fold error (GMFE). These metrics were calculated for the selector output and, for comparison, for each individual mechanistic model and for the oracle assignment.

Predicted–observed agreement was further assessed using the concordance correlation coefficient (CCC) calculated on natural-log-transformed $\:{K}_{p,\mathrm{h}\mathrm{e}\mathrm{a}\mathrm{r}\mathrm{t}}\:$values and reported as logCCC. This metric evaluates concordance with the line of identity, rather than correlation alone, and therefore captures both the strength of the predicted–observed association and systematic deviation from perfect agreement.

For new compounds, the workflow remains unchanged: three $\:{K}_{p,\mathrm{h}\mathrm{e}\mathrm{a}\mathrm{r}\mathrm{t}}$ values are first calculated using the mechanistic models under fixed physiological assumptions, after which the trained selector identifies the method predicted to yield the lowest error and reports the corresponding $\:{K}_{p,\mathrm{h}\mathrm{e}\mathrm{a}\mathrm{r}\mathrm{t}}$ together with all three mechanistic estimates. In this way, the framework is intended to support and standardize mechanistic $\:{K}_{p}$-method selection for further mechanistic modelling of drugs’ pharmacokinetics, rather than relying solely on modeler intuition. All calculations and analyses were performed in R version 4.4.2 (R Core Team, 2024) using readr, dplyr, tidyr, randomForest, xgboost, ggplot2, and a lightweight in-house ridge implementation [29]

## Results

After filtering to compounds with available observed $\:{K}_{p,\mathrm{h}\mathrm{e}\mathrm{a}\mathrm{r}\mathrm{t}}$ values, valid predictions from all three mechanistic models, and calculable corresponding error terms, 55 compounds were retained for selector development and internal evaluation. Oracle-class distribution in the filtered dataset was strongly imbalanced, with 44 compounds assigned to Poulin–Theil, 9 to Rodgers–Rowland, and 2 to Schmitt.

Selector performance was evaluated using repeated stratified 5-fold cross-validation with 10 repetitions, and fold-level performance is summarized in Table 1. Backend differences were modest, and no algorithm showed strong oracle-class recovery. Random forest achieved the highest median balanced accuracy and was therefore selected according to the predefined backend-ranking rule, although Cohen’s κ indicated limited chance-corrected agreement. The remaining backends showed either lower balanced accuracy or greater variability across evaluation folds.

**Table 1:** Cross-validation performance of regression-based selector backends. Performance values are presented as median [minimum–maximum] of fold-level metrics across 50 held-out evaluation folds generated from 10 repetitions of 5-fold cross-validation

Backend	Balanced accuracy*	Cohen’s κ	Accuracy	logMAE	GMFE	Median *N*
Random forest	0.500 [0.333–0.500]	0.000 [−0.222–0.163]	0.818 [0.636–0.889]	0.898 [0.432–1.456]	2.456 [1.541–4.288]	11
Linear regression	0.454 [0.125–0.833]	0.018 [−0.351–0.593]	0.545 [0.222–0.889]	1.252 [0.378–2.937]	3.497 [1.460–18.865]	11
Ridge regression	0.435 [0.148–0.944]	0.060 [−0.305–0.765]	0.556 [0.250–0.917]	1.089 [0.387–2.693]	2.971 [1.473–14.780]	10
XGBoost	0.389 [0.148–0.750]	−0.116 [−0.352–0.621]	0.727 [0.333–0.909]	0.962 [0.466–2.082]	2.617 [1.594–8.024]	11

N denotes the median number of compounds in the held-out evaluation fold

* Balanced accuracy was calculated as the mean recall across oracle classes and was predefined as the primary backend-selection criterion

In terms of numerical agreement between predicted and observed $\:{K}_{p,\mathrm{h}\mathrm{e}\mathrm{a}\mathrm{r}\mathrm{t}}$, median test logMAE was lowest for random forest (0.898), followed by XGB (0.962), RIDGE (1.09), and LM (1.25). Based on the predefined ranking rule, which prioritized median test balanced accuracy against the oracle and used median test Cohen’s $\:\kappa\:\:$as a secondary criterion, random forest was selected as the final backend for prospective application.

A pooled out-of-fold comparison between the selected RF-based workflow and the three mechanistic baselines is shown in Table 2. In this pooled OOF analysis, the RF selector achieved logMAE 0.900, GMFE 2.46, and logCCC 0.377 across 550 held-out predictions. The strongest standalone mechanistic baseline was Poulin–Theil, with slightly better numerical agreement and concordance (logMAE 0.875, GMFE 2.40, logCCC 0.402). Rodgers–Rowland showed weaker agreement (logMAE 1.58, GMFE 4.85, logCCC 0.264), whereas Schmitt underperformed markedly (logMAE 5.09, GMFE 162.17, logCCC − 0.064). As expected, the unattainable oracle benchmark remained best overall (logMAE 0.703, GMFE 2.02, logCCC 0.588).

**Table 2:** Pooled out-of-fold numerical performance of the selected random forest-based selector, the three mechanistic baseline models, and the retrospective oracle benchmark

Model	logMAE	MAE	RMSE	GMFE	logCCC	*N*
Random forest selector	0.900	4.41	9.51	2.46	0.377	550
Poulin–Theil	0.875	4.37	9.50	2.40	0.402	550
Rodgers–Rowland	1.578	39816.22	294567.00	4.85	0.264	550
Schmitt	5.089	5.96	11.06	162.17	−0.064	550
Oracle	0.703	3.91	8.95	2.02	0.588	550

After internal backend selection, the random forest selector was refit on the full internal dataset and applied prospectively to the transthyretin stabilisers. For both tafamidis and acoramidis, the selector chose Poulin–Theil as the preferred mechanistic method. The resulting selected $\:{K}_{p,\mathrm{h}\mathrm{e}\mathrm{a}\mathrm{r}\mathrm{t}}$ estimates were 5.86 for tafamidis and 0.593 for acoramidis.

**Fig. 2:**
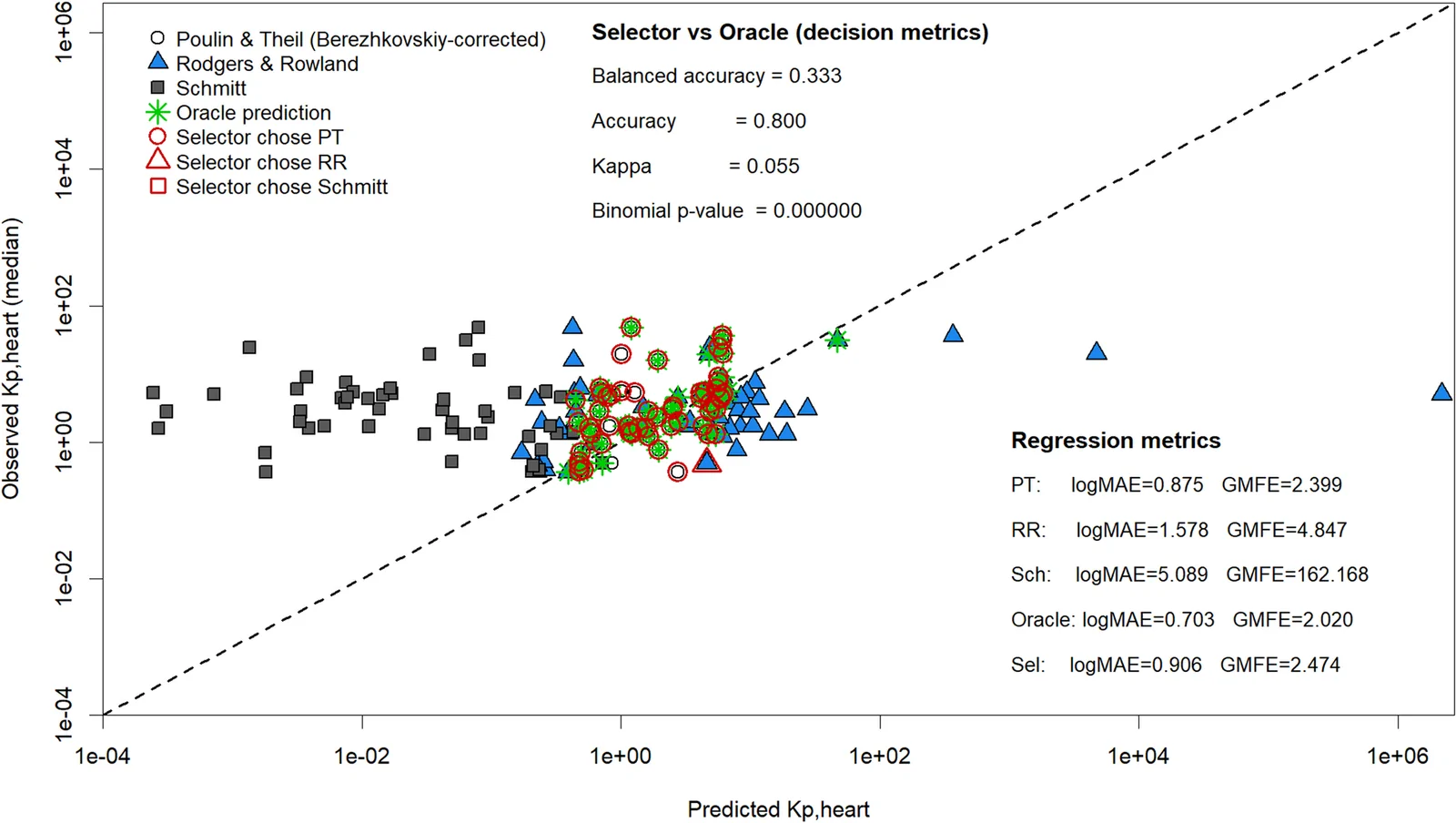
Predicted versus observed $\:{K}_{p,heart}$ values using one representative selector assignment per compound, derived from repeated out-of-fold predictions across stratified 5-fold cross-validation (10 repetitions). Selector-assigned predictions are highlighted in red, and oracle predictions are shown in green

Selector choices were dominated by Poulin–Theil, with occasional Rodgers–Rowland and rare Schmitt selections, reflecting the skewed distribution of oracle-best labels Fig. 2 presents all three mechanistic predictions together with one representative selector-assigned value per compound, derived from repeated cross-validation evaluations.

**Fig. 3:**
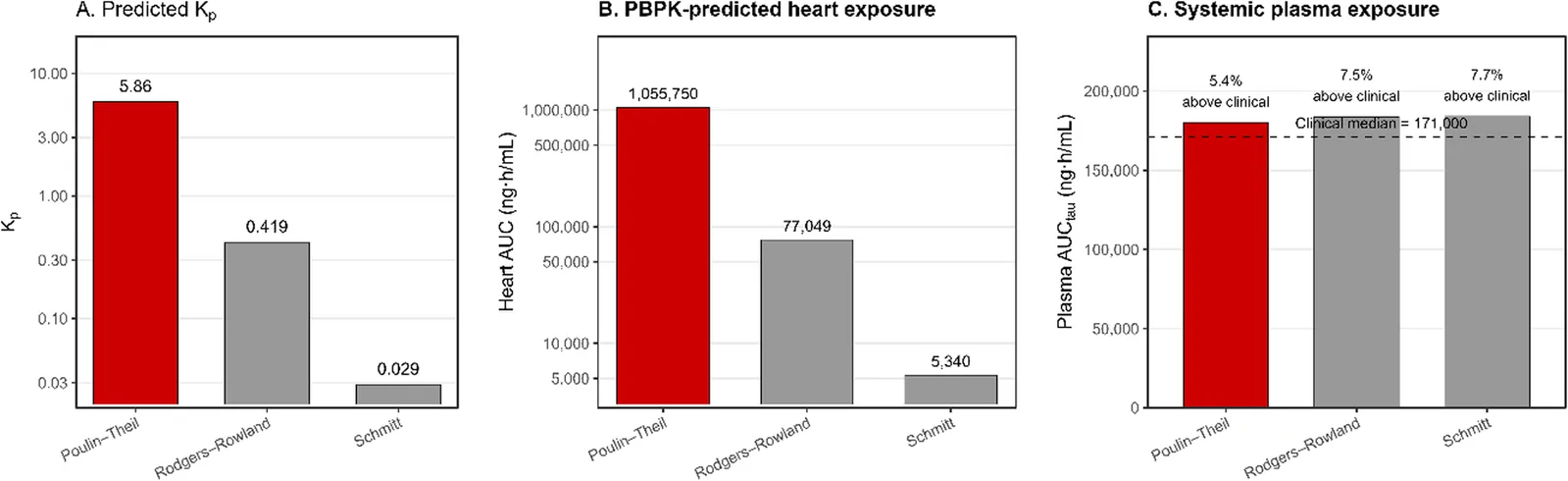
Prospective PBPK use-case analysis of alternative $\:{K}_{p,heart}$ estimates. Poulin–Theil, Rodgers–Rowland, and Schmitt $\:{K}_{p,heart}$ values were propagated into the tafamidis PBPK model, and AUC values were calculated over the day-7 dosing interval from 144 to 168 h after treatment initiation. Panels show: (A) $\:{K}_{p,heart}$ values, (B) PBPK-predicted heart $\:AU{C}_{\tau\:}$, and (C) systemic plasma $\:AU{C}_{\tau\:}$. The dashed line in panel C denotes the observed clinical trial median plasma $\:AU{C}_{\tau\:}$ of 171,000 ng·h/mL

In the tafamidis PBPK use case, the three alternative $\:{K}_{p,\mathrm{h}\mathrm{e}\mathrm{a}\mathrm{r}\mathrm{t}}$ estimates produced markedly different predicted cardiac exposures during the day-7 dosing interval (144–168 h), while systemic plasma $\:AU{C}_{\tau\:}$ remained similar across simulations. This pattern is consistent with the heart being modelled as a distribution-only compartment, where $\:{K}_{p,\mathrm{h}\mathrm{e}\mathrm{a}\mathrm{r}\mathrm{t}}$ directly determines tissue partitioning but does not introduce local clearance. A comparison of AUC values obtained using different $\:{K}_{p,\mathrm{h}\mathrm{e}\mathrm{a}\mathrm{r}\mathrm{t}}$ estimates is shown in Fig. 3.

## Discussion

All three models used for $\:{K}_{p}$ prediction and evaluated in this study are based on physiologically and biochemically motivated principles and have previously been used for tissue-distribution estimation. However, determining which model should be preferred for predicting xenobiotic partitioning from plasma to a specific human organ remains unresolved. This difficulty reflects the scarcity of curated human tissue-partitioning data and the lack of a universally dominant mechanistic approach across chemical space. By relying on experimentally determined human $\:{K}_{p,\mathrm{h}\mathrm{e}\mathrm{a}\mathrm{r}\mathrm{t}}\:$values and restricting evaluation to the human heart setting, the present work provides a human-centered benchmark for method comparison.

A major consequence of the current workflow is that selector performance is interpreted through repeated internal cross-validation, rather than through a single train/test split. This is important because the earlier single-split framework tested by us proved unstable with respect to backend ranking. Under the repeated stratified cross-validation design, random forest emerged as the best-performing selector backend according to the predefined criterion, whereas the earlier split-based workflow had favoured different model. This indicates that backend choice was sensitive to resampling design and confirms that repeated internal evaluation is more appropriate for a dataset of this size.

The selector results should be interpreted primarily through balanced accuracy and Cohen’s $\:\kappa\:$, rather than raw accuracy alone. The median balanced accuracy of 0.500 for random forest means that, after averaging recall equally across the three mechanistic classes, the selector correctly recovered about half of the oracle-assigned compounds. In the present imbalanced setting, this is more informative than overall accuracy because it reduces the apparent advantage of strategies dominated by the majority PT class. By contrast, the relatively high median random forest accuracy of 0.818 could easily be overinterpreted if considered alone, because a selector that frequently defaults to PT will appear accurate when PT dominates the oracle distribution.

This interpretation is reinforced by the $\:\kappa\:$ values. Cohen’s $\:\kappa\:\:$measures agreement beyond that expected by chance, taking class prevalence into account. The fact that median $\:\kappa\:\:$values remained low for all backends, including 0 for random forest, indicates that much of the apparent agreement was still driven by oracle-class imbalance. In practical terms, random forest performed best under the predefined class-balanced ranking criterion, but its decisions remained strongly constrained by the predominance of the PT class. The slightly higher median $\:\kappa\:$ values for RIDGE and LM suggest some additional chance-corrected structure, but not enough to overcome their weaker balanced accuracy.

The oracle benchmark should itself be interpreted cautiously. It is not a prospective predictor, but a retrospective upper bound that identifies which mechanistic method happened to be least wrong after the observed $\:{K}_{p,\mathrm{h}\mathrm{e}\mathrm{a}\mathrm{r}\mathrm{t}}\:$value is already known. Accordingly, the gap between the selector and the oracle reflects remaining room for improvement in method selection, not a realistic target that can be achieved directly in practice.

The pooled OOF comparison clarifies the practical role of the selector. The random forest selector achieved logMAE 0.900 and GMFE 2.46, meaning that selected $\:{K}_{p,\mathrm{h}\mathrm{e}\mathrm{a}\mathrm{r}\mathrm{t}}\:$values differed from observations by about 2.5-fold on average. However, the strongest standalone mechanistic baseline, Poulin–Theil, performed slightly better numerically, with logMAE 0.875 and GMFE 2.40. Thus, under the present dataset and implementation assumptions, the selector did not materially outperform the best baseline in terms of absolute numerical error.

This should not be interpreted as a failure of the approach. Rather, it defines its actual value in the current setting. The selector’s main contribution is not a large reduction in $\:{K}_{p,\mathrm{h}\mathrm{e}\mathrm{a}\mathrm{r}\mathrm{t}}\:$error relative to the strongest mechanistic model, but the provision of a transparent, reproducible, and data-driven rule for method choice. When several mechanistic methods are plausible, the selector formalizes a decision that would otherwise often rely on modeler intuition. In that sense, its practical role is to support and standardize mechanistic method selection rather than to replace the mechanistic models themselves.

It is also instructive to contextualize the present framework within broader efforts to optimize tissue distribution predictions. For instance, when preclinical pharmacokinetic data are available, several groups have demonstrated that optimizing simplified PBPK models or deriving empirical tissue scaling factors from animal models (e.g., rats, dogs, and monkeys) can significantly improve the accuracy of human volume of distribution and drug disposition estimates [30, 31]. Other strategies bypass in vivo animal testing altogether by utilizing protein binding assays to correct theoretically calculated partition coefficients, facilitating earlier human pharmacokinetic predictions [32]. Furthermore, pure machine learning and quantitative structure-activity relationship (QSAR) methods, such as repeated random forest algorithms, have been effectively deployed to directly predict tissue $\:{K}_{p}$ values and handle missing data matrices across multiple organs [33]. While these approaches emphasize cross-species translation, in vitro-to-in vivo extrapolation, or direct empirical parameter prediction, the workflow proposed here occupies a distinct space. By restricting the learning environment entirely to human data and keeping the underlying mechanistic equations intact, our approach uses pattern recognition not to predict the partitioning endpoint directly, but exclusively to formalize the decision-making process among competing mechanistic models in a target-specific context.

The strong dominance of Poulin–Theil in the oracle distribution is itself informative. It suggests that, under the fixed physiological assumptions used here, PT is a strong default model for human whole-heart $\:{K}_{p}$ prediction. This conclusion is also consistent with the prospective stabiliser application, in which the selector chose PT for both tafamidis and acoramidis. However, this same dominance also constrains learning: when 44 of 55 compounds are oracle-assigned to PT, there is limited information available to define the conditions under which Rodgers–Rowland or Schmitt become preferable.

The PBPK example should therefore be interpreted as a translational illustration of the relevance of $\:{K}_{p,\mathrm{h}\mathrm{e}\mathrm{a}\mathrm{r}\mathrm{t}}$ selection, rather than as an independent validation of the selector. In a whole-body PBPK model, tissue-to-plasma partition coefficients determine how systemic exposure is distributed across organs; consequently, uncertainty in $\:{K}_{p,\mathrm{h}\mathrm{e}\mathrm{a}\mathrm{r}\mathrm{t}}$ propagates directly into predicted cardiac exposure. This is particularly relevant for tafamidis, where cardiac exposure is pharmacologically meaningful in the context of transthyretin amyloid cardiomyopathy, even though the present heart compartment does not include local clearance or a cardiac pharmacodynamic feedback mechanism. Thus, the PBPK application demonstrates how method choice at the $\:{K}_{p}$ prediction stage can affect downstream organ-level exposure estimates used in mechanistic pharmacokinetic modelling.

Several limitations affect interpretation. First, the benchmark dataset remains small, so repeated cross-validation improves internal use of the available data but does not eliminate uncertainty arising from limited sample size. This data scarcity is a common challenge for models relying strictly on public domain information. While highly predictive QSAR models for tissue distribution have been successfully developed for other organs, such as the model for unbound brain partitioning by Dolgikh et al., which leveraged a massive, in-house proprietary dataset of approximately 1000 compounds, researchers modeling human cardiac exposure without access to closed pharmaceutical databases must operate under severe data constraints [34]. In such data-poor environments, our hybrid approach of using machine learning merely to select among established mechanistic models, rather than fitting a purely empirical model from scratch, provides a necessary and pragmatic solution.

Second, class imbalance is substantial, which makes minority-class recall estimates unstable and depresses chance-corrected agreement.

Third, physiological and tissue-composition values were fixed across the mechanistic calculators according to their original publications. As elegantly demonstrated by Utsey et al., the use of differing tissue composition data can confound the comparative evaluation of mechanistic $\:{K}_{p}$ models and significantly impact predictive performance [35]. While Utsey et al. proposed a standardized tissue composition to control for this variability across multiple organs, we elected to evaluate each model using the specific composition parameters reported by its original authors. Consequently, models that are particularly sensitive to specific inputs, such as phospholipid terms, pH partitioning, extracellular protein assumptions, or membrane-partition surrogates, may be inherently advantaged or disadvantaged by their native physiological assumptions.

Fourth, the prospective application to stabilisers demonstrates workflow use, but it does not constitute external validation.

The especially weak performance of Schmitt warrants cautious interpretation. In principle, Schmitt is the most explicitly compositional of the three frameworks. In the present implementation, however, it depends on several practically necessary assumptions, including treatment of the acidic phospholipid contribution and the use of lipophilicity-derived surrogate input when direct neutral phospholipid partition data are unavailable. Poor performance under these conditions therefore does not necessarily invalidate the Schmitt framework itself but rather suggests that it may be particularly sensitive to parameter quality and implementation choices in the human heart setting.

The conceptual strength of the proposed workflow lies in its hybrid structure. The mechanistic equations remain intact and physiologically interpretable, while a data-driven layer provides empirical guidance on method selection. This preserves mechanistic transparency while reducing reliance on subjective method choice. In the present dataset, that strategy produced meaningful internal discrimination beyond uninformed guessing, but it did not overcome the dominance of the strongest baseline to a large numerical extent.

Overall, when multiple mechanistic tissue-partitioning methods are available, a regression-based selector offers a transparent and reproducible rule for choosing among them. Under the current human whole-heart benchmark, Poulin–Theil remained the strongest standalone baseline and random forest the best empirical selector backend. The principal limitation is therefore not algorithmic complexity, but benchmark structure: the limited size and strong class imbalance of the available human $\:{K}_{p,\mathrm{h}\mathrm{e}\mathrm{a}\mathrm{r}\mathrm{t}}$ dataset. Expansion of the human benchmark dataset, together with future incorporation of physiological variability and improved descriptor representation of phospholipid- and pH-dependent partitioning, represents the most direct path toward improving selector robustness and broader utility.

## Conclusion

In conclusion, the proposed hybrid mechanistic–AI/ML workflow provides a reproducible framework for selecting heart $\:{K}_{p}$ prediction methods on a compound-specific basis. Although Poulin–Theil remained the strongest standalone model in the current analysis, the selector formalized method choice and demonstrated how uncertainty in $\:{K}_{p,heart}$ estimation can propagate into PBPK-predicted cardiac exposure. Further improvement of this approach will require broader, higher-quality, and more diverse human heart distribution data to better characterize the conditions under which different mechanistic models are preferable.

## Supplementary Information

Below is the link to the electronic supplementary material.

Supplementary Material 1 (DOCX 23.5 KB)

Supplementary Material 2 (CSV 14.9 KB)

Supplementary Material 3 (R 48.4 KB)

Supplementary Material 4 (DOCX 31.5 KB)

## Data Availability

The dataset and descriptor files used in this study are provided as supplementary files. These include the curated human heart-to-plasma partition coefficient dataset, compound descriptors used for selector development, and files required to reproduce the mechanistic Kp calculations and AI/ML-based model-selection analysis.
