# Supplementary material for: Formalizing method choice: a data-driven selection of mechanistic models for predicting human heart drug partitioning
Source: J Pharmacokinet Pharmacodyn. 2026 Sep 25;53(6):56. doi: 10.1007/s10928-026-10057-4 (PMC13615157; doi:10.1007/s10928-026-10057-4)
Supplement: Supplementary file 1 [file 10928_2026_10057_MOESM1_ESM.docx]

Supplementary Table 1 Physiological data used for models

| Parameter | Code variable | Value | Compartment | Meaning |
| --- | --- | --- | --- | --- |
| Heart intracellular pH | pH_heart | 7.10 | Heart | Myocyte pH |
| Plasma pH | pH_plasma | 7.40 | Plasma | Blood pH |
| Extracellular water (heart) | EW_heart | 0.313 | Heart | ECF fraction |
| Intracellular water (heart) | IW_heart | 0.445 | Heart | ICF fraction |
| Neutral lipids (heart) | NL_heart | 0.0115 | Heart | Neutral lipid fraction |
| Neutral phospholipids (heart) | NP_heart | 0.0166 | Heart | Neutral phospholipid fraction |
| Extracellular water (plasma) | EW_plasma | 0.945 | Plasma | Water fraction |
| Neutral lipids (plasma) | NL_plasma | 0.0023 | Plasma | Neutral lipids |
| Neutral phospholipids (plasma) | NP_plasma | 0.0013 | Plasma | Neutral phospholipids |
| Heart albumin scaling | R_ALB_heart | 0.157 | Heart | Heart-to-plasma albumin ratio |
| Charge permeability floor | alpha_charge | 1e-3 | — | Minimum neutral fraction |
| Heart cell water | CELL_W_heart | 0.70 | Heart cells | Cytosolic water fraction |
| Heart cell lipids | CELL_L_heart | 0.074 | Heart cells | Total cellular lipid fraction |
| Heart cell protein | CELL_P_heart | 0.26 | Heart cells | Cellular protein fraction |
| Fraction interstitial (heart) | F_I_heart | 0.14 | Heart | Interstitial volume fraction |
| Plasma water (Schmitt) | F_W_Plasma | 0.928 | Plasma | Fractional water for Schmitt |
| Plasma protein (Schmitt) | F_P_Plasma | 0.070 | Plasma | Fractional protein for Schmitt |
| Lipid neutral fraction | L_FRAC_NL | 0.48 | Heart cell lipids | Neutral lipids % |
| Lipid neutral phospholipids | L_FRAC_NPL | 0.43 | Heart cell lipids | Neutral PL % |
| Lipid acidic phospholipids | L_FRAC_APL | 0.09 | Heart cell lipids | Acidic PL % |
