# Supplementary material for: Formalizing method choice: a data-driven selection of mechanistic models for predicting human heart drug partitioning
Source: J Pharmacokinet Pharmacodyn. 2026 Sep 25;53(6):56. doi: 10.1007/s10928-026-10057-4 (PMC13615157; doi:10.1007/s10928-026-10057-4)
Supplement: Supplementary file 2 [file 10928_2026_10057_MOESM4_ESM.docx]

Supplementary Table 1 Physiological data used for models

| Parameter | Code variable | Value | Compartment | Meaning | Source |
| --- | --- | --- | --- | --- | --- |
| Heart intracellular pH | pH_heart | 7.10 | Heart | Myocyte pH | [1] |
| Plasma pH | pH_plasma | 7.40 | Plasma | Blood pH | [2] |
| Extracellular water (heart) | EW_heart | 0.313 | Heart | ECF fraction | [2]  Derived from total heart water 0.758 split according to Rodgers 2005 EW/IW proportions |
| Intracellular water (heart) | IW_heart | 0.445 | Heart | ICF fraction | [2]  Derived from total heart water 0.758 split according to Rodgers 2005 EW/IW proportions |
| Neutral lipids (heart) | NL_heart | 0.0115 | Heart | Neutral lipid fraction | [3] |
| Neutral phospholipids (heart) | NP_heart | 0.0166 | Heart | Neutral phospholipid fraction | [4] |
| Extracellular water (plasma) | EW_plasma | 0.945 | Plasma | Water fraction | [3] |
| Neutral lipids (plasma) | NL_plasma | 0.0023 | Plasma | Neutral lipids | [5] |
| Neutral phospholipids (plasma) | NP_plasma | 0.0013 | Plasma | Neutral phospholipids | [5] |
| Heart albumin scaling | R_ALB_heart | 0.157 | Heart | Heart-to-plasma albumin ratio | [5] |
| Charge permeability floor | alpha_charge | 1e-3 | — | Minimum neutral fraction | [1] |
| Heart cell water | CELL_W_heart | 0.70 | Heart cells | Cytosolic water fraction | [1] |
| Heart cell lipids | CELL_L_heart | 0.074 | Heart cells | Total cellular lipid fraction | [1] |
| Heart cell protein | CELL_P_heart | 0.26 | Heart cells | Cellular protein fraction | [1] |
| Fraction interstitial (heart) | F_I_heart | 0.14 | Heart | Interstitial volume fraction | [1] |
| Plasma water (Schmitt) | F_W_Plasma | 0.928 | Plasma | Fractional water for Schmitt | [1] |
| Plasma protein (Schmitt) | F_P_Plasma | 0.070 | Plasma | Fractional protein for Schmitt | [1] |
| Lipid neutral fraction | L_FRAC_NL | 0.48 | Heart cell lipids | Neutral lipids % | [1] |
| Lipid neutral phospholipids | L_FRAC_NPL | 0.43 | Heart cell lipids | Neutral PL % | [1] |
| Lipid acidic phospholipids | L_FRAC_APL | 0.09 | Heart cell lipids | Acidic PL % | [1] |

[1]	W. Schmitt, “General approach for the calculation of tissue to plasma partition coefficients,” *Toxicol. In Vitro*, vol. 22, no. 2, pp. 457–467, Mar. 2008, doi: 10.1016/j.tiv.2007.09.010.

[2]	T. Rodgers, D. Leahy, and M. Rowland, “Physiologically Based Pharmacokinetic Modeling 1: Predicting the Tissue Distribution of Moderate-to-Strong Bases,” *J. Pharm. Sci.*, vol. 94, no. 6, pp. 1259–1276, Jun. 2005, doi: 10.1002/jps.20322.

[3]	P. Poulin and F. Theil, “*A Priori* Prediction of Tissue:Plasma Partition Coefficients of Drugs to Facilitate the Use of Physiologically‐Based Pharmacokinetic Models in Drug Discovery,” *J. Pharm. Sci.*, vol. 89, no. 1, pp. 16–35, Jan. 2000, doi: 10.1002/(SICI)1520-6017(200001)89:1<16::AID-JPS3>3.0.CO;2-E.

[4]	P. Poulin and F. P. Theil, “Prediction of pharmacokinetics prior to in vivo studies. II. Generic physiologically based pharmacokinetic models of drug disposition,” *J Pharm Sci*, vol. 91, no. 5, pp. 1358–1370, 2002, doi: 10.1002/jps.10128.

[5]	T. Rodgers and M. Rowland, “Physiologically based pharmacokinetic modelling 2: Predicting the tissue distribution of acids, very weak bases, neutrals and zwitterions,” *J. Pharm. Sci.*, vol. 95, no. 6, pp. 1238–1257, Jun. 2006, doi: 10.1002/jps.20502.
